# A Flow Stress Model of 300M Steel for Isothermal Tension

**DOI:** 10.3390/ma14020252

**Published:** 2021-01-07

**Authors:** Rongchuang Chen, Shiyang Zhang, Xianlong Liu, Fei Feng

**Affiliations:** 1 School of Materials Science & Engineering, Hubei University of Automotive Technology, Shiyan 442002, China; 202011104@huat.edu.cn (S.Z.); liuxianlong@huat.edu.cn (X.L.); 2 State Key Laboratory of Materials Processing and Die & Mould Technology, Huazhong University of Science and Technology, Wuhan 430074, China; fengfei@hust.edu.cn; 3 School of Mechanical & Electrical Engineering, Wuhan Institute of Technology, Wuhan 430205, China

**Keywords:** flow stress model, tensile deformation, constitutive model, stress correction

## Abstract

To investigate the effect of hot working parameters on the flow behavior of 300M steel under tension, hot uniaxial tensile tests were implemented under different temperatures (950 °C, 1000 °C, 1050 °C, 1100 °C, 1150 °C) and strain rates (0.01 s^−1^, 0.1 s^−1^, 1 s^−1^, 10 s^−1^). Compared with uniaxial compression, the tensile flow stress was 29.1% higher because dynamic recrystallization softening was less sufficient in the tensile stress state. The ultimate elongation of 300M steel increased with the decrease of temperature and the increase of strain rate. To eliminate the influence of sample necking on stress-strain relationship, both the stress and the strain were calibrated using the cross-sectional area of the neck zone. A constitutive model for tensile deformation was established based on the modified Arrhenius model, in which the model parameters (*n*, *α*, *Q*, ln(*A*)) were described as a function of strain. The average deviation was 6.81 MPa (6.23%), showing good accuracy of the constitutive model.

## 1. Introduction

The 300M steel (yield strength ≥ 1800 MPa), a kind of low-alloyed ultra-high strength steel, is an important structural material used for large parts in aircrafts, ships, and nuclear power plants. To improve service performance, those large structural parts are often hot forged. However, in the forming of large parts, folding defects usually occur due to the failure of precise material control because of inaccurate flow stress prediction. Thus, establishment of an accurate flow stress model is a key issue in forging.

The understanding of the effects of hot working parameters (e.g., forging temperature, strain rate, strain) on flow behavior is vital for precise constitutive modelling. The strain rate and temperature effects were investigated by Ghavam et al. [1] and Huang et al. [2], and tensile flow stress models for IMI834 titanium alloy and 42CrMo steel were proposed. Lin et al. [3] constructed a phenomenological model to describe the influence of hot working parameters on flow stress in hot tension of Al-Cu-Mg alloy. Besides, the microstructure evolution (e.g., average dislocation density, average grain size, damage) plays an important role in flow stress evolution. The material flow behavior of 304HCu stainless steel under various temperatures and strain rates in tensile deformation was investigated by Yadav et al. [4], and a tensile flow stress model considering the evolution of mobile and forest dislocations was established. The grain evolution of C-Mn steel in hot tensile deformation was studied by Dolzhenko et al. [5], and result showed the average grain size and tensile yield stress followed the Hall-Petch relationship. Moreover, an accurate flow stress model should eliminate the experimental error due to necking in tension. In the hot tension experiment of Murata et al. [6], the necking image of notched specimen was recorded, and the flow stress–strain curve of SS400 steel was corrected with the help of image analysis and inverse analysis of finite element simulation. By a similar technique, Zhao et al. [7] successfully calibrated the flow stress of Q195 steel, HSLA350 aluminum alloy, and AL6061 aluminum alloy. Gain et al. [8] proposed a flow stress model of AlSi9Cu3 alloy taking into account the stress triaxiality and damage evolution, and the model was applicable in various stress states (uniaxial tension, uniaxial compression, and Astakhov test). Until now, precise modelling of tensile flow stress is still facing difficulty due to the lack of knowledge about how stress states affect flow behaviors of metal materials.

Specifically, for 300M steel, a dislocation–based constitutive model considering dynamic, meta–dynamic, and static recrystallization in both single and multiple pass compression has been established by our group [9,10,11]. The softening behavior of 300M steel between passes was investigated by Liu et al., and models quantifying the meta–dynamic [12] and static recrystallization softening [13] were built. Recently, the fracture behavior of 300M steel in tensile deformation was studied by Wen et al. [14], but the tensile flow stress model of 300M steel has not been established so far.

Accordingly, as an essential part of precise prediction of flow stress and microstructure evolution of 300M steel in high temperature deformation, the present research aims to establish an accurate model to describe the flow stress evolution in tension. The flow stress and logarithmic strain will be corrected using the minimum area in the necking zone of specimen. A tensile flow stress model will be constructed.

## 2. Materials and Experiments

**Figure 1:**
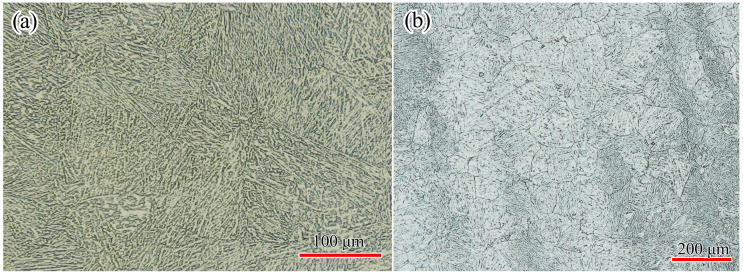
Microstructure of 300M steel in (**a**) as-received state, and (**b**) after tempering at 560 °C for 2 h.

The 300M steel ingot (Φ300 mm × 1000 mm) was received in the as-forged state from China Erzhong Group Cooperation (Deyang, China). The chemical composition (weight percentage) was 93.982Fe-2.562Si-0.896Cr-0.824Ni-0.808Mn-0.435Mo-0.39C-0.086V-0.017S. All samples used in this research were taken from the half radius of the ingot. The initial microstructure was martensite (Figure 1a). In order to show the original austenite grain boundaries, the samples were tempered at 560 °C for 2 h, polished according to the standard metallographic procedure, and etched in the solution (1.7% hydrochloric acid, 22% detergent, 22% carbon tetrachloride, and balanced saturated picric acid) [15]. The microstruture was tempered martensite after tempering (Figure 1b), and the initial average grain size was 37.5 μm.

Hot tensile tests were carried out on a thermal deformation simulator (Gleeble 3500, Dynamic Systems Inc., New York, NY, USA) at five temperatures (950 °C, 1000 °C, 1050 °C, 1100 °C, 1150 °C) and four speeds (0.174 mm/s, 1.74 mm/s, 17.4 mm/s, 174 mm/s). The temperature range corresponded to the usual hot working temperature of this material. The deformation speed corresponded to 0.01 s^−1^, 0.1 s^−1^, 1 s^−1^, and 10 s^−1^, respectively. In order to control the position of the necking zone, a notch (Φ8 mm × 12 mm) was turned in the middle of the specimen. A dilatometer was clipped in the middle of the specimen to measure the neck diameter. The force and elongation of specimens were automatically measured by the machine. During the experiment, specimen was electrically heated, and a thermal couple welded in the notching area was able to measure the specimen temperature and transfer the data to a computer. The heating power could be automatically adjusted by a computer program to obtain specific temperatures.

**Figure 2:**
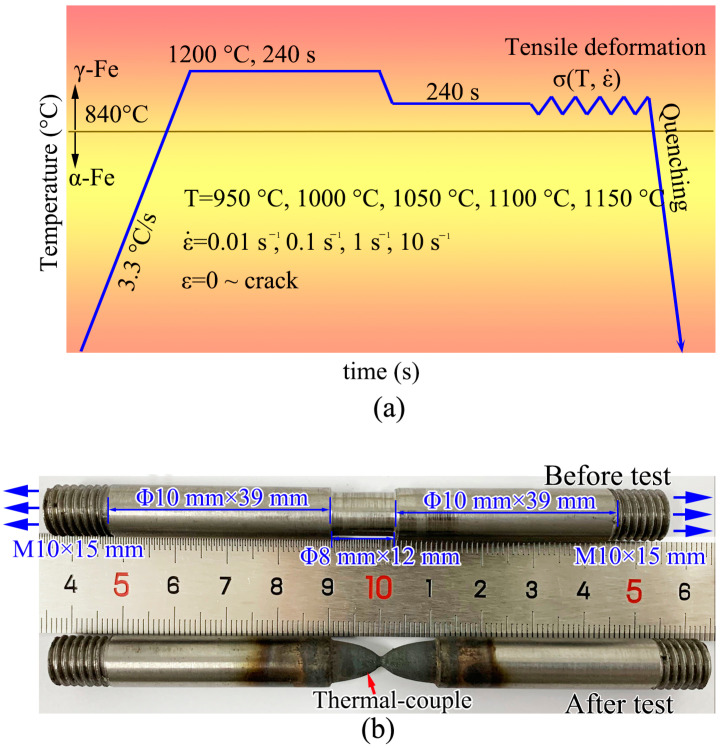
Experimental procedure. (**a**) thermal-mechanical process, (**b**) specimen photos before and after deformation.

The thermal-mechanical process is shown in Figure 2a. The test sample was heated at a heating rate of 200 °C/min to 1200 °C, held at 1200 °C for 4 min to complete austenization, and cooled to deformation temperature for another holding of 4 min. In total, twenty tests were carried out, and the experimental parameters are shown in Table 1. Once the specimen cracked, test was ceased and specimen was water quenched. The specimen photos are shown in Figure 2b. It should be noted that the length of the sample deformation area was 12 mm, and the chamfer length was 1 mm.

## 3. Results and Discussion

### 3.1. Force-Stroke Curve

**Figure 3:**
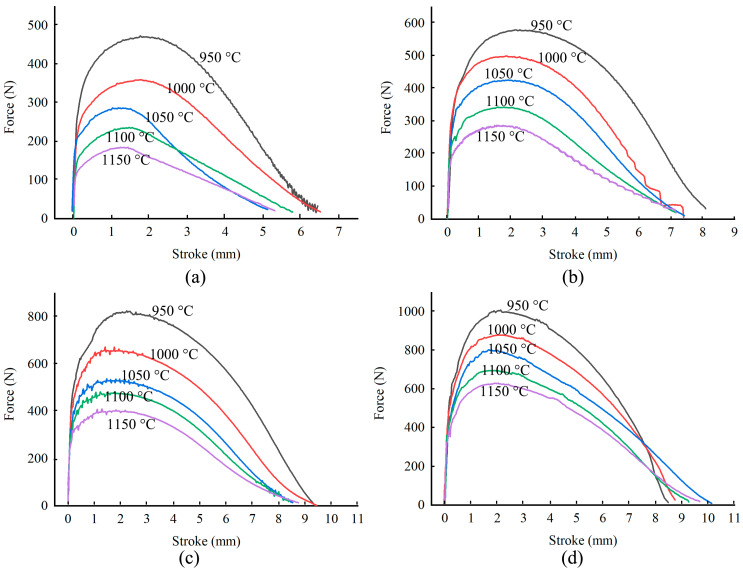
Force–stroke curve during tensile test. (**a**) 0.01 s^−1^, (**b**) 0.1 s^−1^, (**c**) 1 s^−1^, (**d**) 10 s^−1.^

The force-stroke curves obtained in the tensile tests are shown in Figure 3. The deformation process was divided into four stages: the elastic stage, the stable deformation stage, the necking stage, and the fracture stage. Force increased linearly as the stroke increased in the elastic stage. When the strain exceeded the elastic limit, the stable deformation stage began. Since the strain was too low to trigger dynamic recrystallization, only work-hardening and dynamic recovery occurred. Under a high temperature and a low strain rate, the peak force was low, because dislocation annihilation was more complete. As the stroke increased further, necking gradually appeared. The loading force decreased due to the combing effect of the reduction of cross-sectional area and the dynamic recrystallization softening. In order to eliminate the influence of necking on the flow behavior in tensile deformation, the true stress and the logarithmic strain could be corrected by measuring the cross-sectional area of the necking zone of the specimen. In the fracture stage, micro-void formed and grew near the necking zone, leading to breakage [16]. It can be seen that the ultimate elongation was greater under a higher strain rate and at a lower temperature, and the cross-sectional area of the specimen slowly reduced to zero, indicating that the ductile fracture occurred.

### 3.2. Stress and Strain Correction

The true stress (*σ*) was defined by:

(1) $$\sigma =\frac{F}{A}$$

Here, *F* was the loading force (N), and *A* was the cross-section area (m^2^). In the present investigation, *F* was the tensile force, and *A* was the minimum cross-sectional area of the necking zone of the specimen. The cross-section of the specimen in this test was round, so *A* = π*d*^2^/4, where d was the minimum cross-sectional diameter (m). The value of *F* was an exported data of the experiment equipment. The value of *d* was measured by a dilatometer.

The logarithmic strain, *ε*, was calculated by:

(2) $$\varepsilon =\ln\left(\frac{l}{l_{0}}\right)$$

Here, *l*_0_ was the initial gauge length, and *l* was the gauge length after deformation. In this investigation, considering that the sample volume remained constant, *l*/*l*_0_ equaled *A*_0_/*A*, so:

(3) $$\varepsilon =\ln\left(\frac{A_{0}}{A}\right)$$

Here, *A*_0_ and *A* were calculated by the cross-sectional diameter of the specimen. 

**Figure 4:**
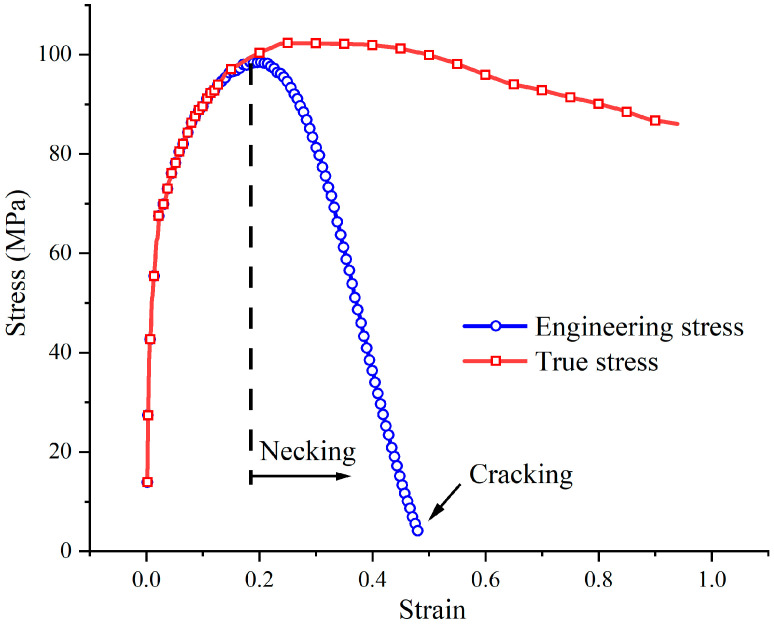
Comparison of engineering stress and true stress of 300M steel under 1050 °C 0.1 s^−1^.

Strains and stresses were calculated according to Equations (1) and (3). The comparison of engineering stress and true stress is shown in Figure 4. In the elastic stage and the stable deformation stage, the engineering stress curve and the true stress curve were almost the same. But in the necking stage, an obvious difference of the two curves was shown.

### 3.3. Tensile Flow Behavior

**Figure 5:**
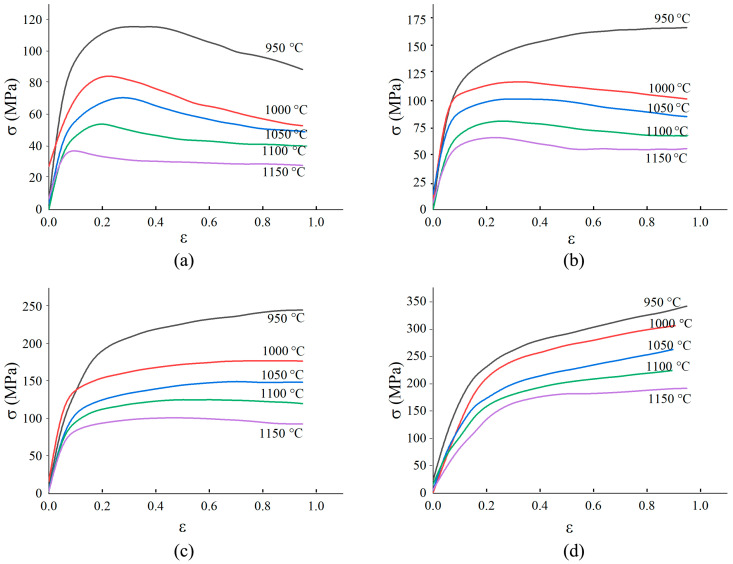
Tensile flow stress of 300M steel under: (**a**) 0.01 s^−1^, (**b**) 0.1 s^−1^, (**c**) 1 s^−1^, (**d**) 10 s^−1^.

**Figure 6:**
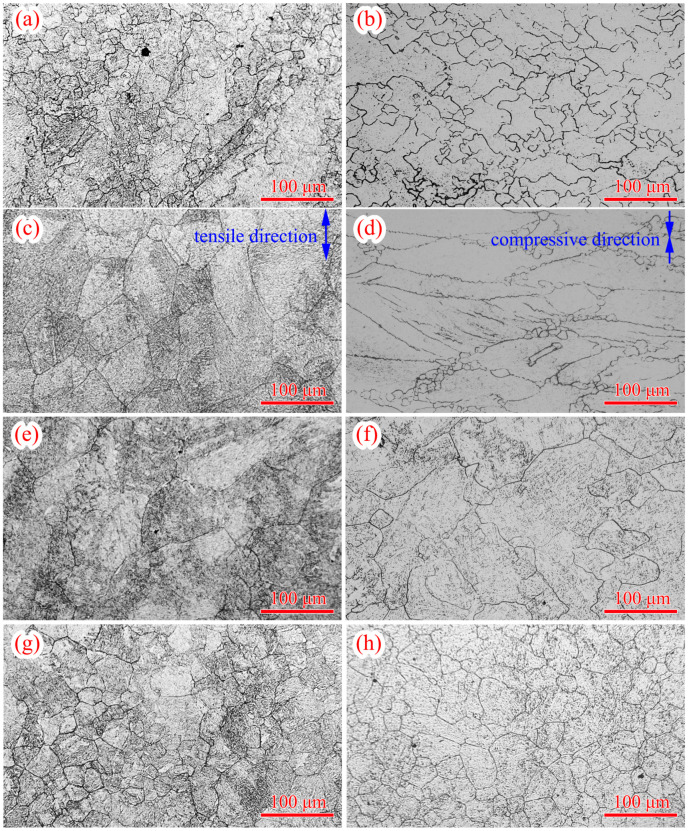
Microstructures of 300M steel after tension at (**a**) 950 °C 0.01 s^−1^, (**c**) 950 °C 10 s^−1^, (**e**) 1150 °C 0.01 s^−1^, (**g**) 1150 °C 10 s^−1^, and after compression at (**b**) 950 °C 0.01 s^−1^, (**d**) 950 °C 10 s^−1^, (**f**) 1150 °C 0.01 s^−1^, (**h**) 1150 °C 10 s^−1^.

The true stress-logarithmic strain curves of 300M steel calculated by the above method are shown in Figure 5. Under a high temperature and a low strain rate, the true stress was low due to a low dislocation motion barrier. This phenomenon was also found in the compression of 300M steel [11], GH4169 alloy [17], GH4698 alloy [18], etc. The previous result in Figure 3, that the ultimate elongation was greater under a higher strain rate and a lower temperature, could be interpreted by the flow stress curves in Figure 5. Under a higher strain rate, work-hardening-shaped stress-strain curve was obtained, and the work-hardening led to the strengthening of the necking zone, causing deformation of the adjacent zones of specimen, and making the ultimate elongation greater. The flow stress curve shapes gradually transited from single peaked to exponential hardened when the strain rate increased. This was because the dynamic recrystallization was more easily to complete at a lower strain rate, while work-hardening played a more important role at a higher strain rate. Compared with Figure 6c, much more small recrystallized grains were found in Figure 6a. But only a few small recrystallized grains could be seen in Figure 6e, because under a low strain rate (0.01 s^−1^) and high temperature (1150 °C), small recrystallized grains gradually coarsened. The grains in Figure 6g were relatively small because the deformation time was short, and the grains did not have enough time to grow [19]. 

**Figure 7:**
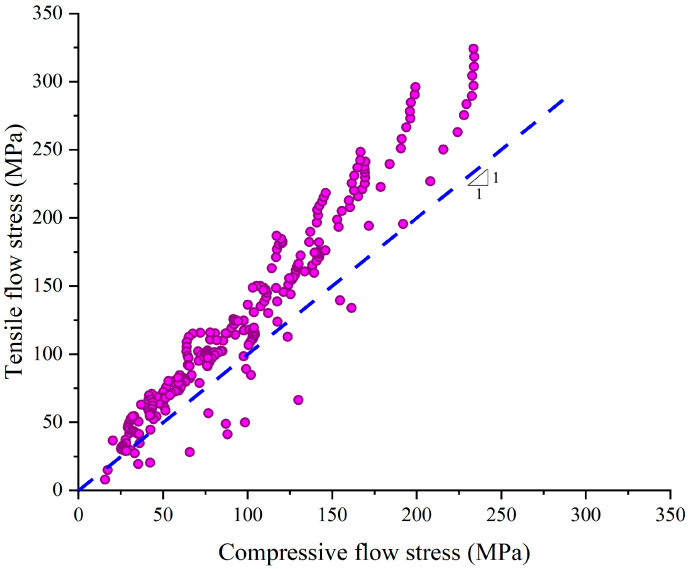
Comparison of tensile and compressive flow stress.

Comparing the tensile flow stresses with the compressive flow stresses, it was found that the tensile flow stresses were 29.1% (27.8 MPa) greater, as shown in Figure 7. The flow stress difference could be explained by: (a) the flow stress was influenced by the stress state [8]. (b) the dynamic recrystallization in the tension was less sufficient, and the dynamic recrystallization softening was weakened by the work-hardening, resulting in a higher flow stress. This could be demonstrated by the comparison of the microstructures of 300M steel after tension and after compression. It could be seen in Figure 6 that more small recrystallized grains could be found after compression. Thus, the compressive flow stress model could not be applied in the tensile deformation of 300M steel, and it was necessary to establish the constitutive model for the high temperature tension of 300M steel.

### 3.4. Constitutive Model

Some constitutive models are frequently used in modeling of steels, for example, the Johnson-Cook model [20], the Zerilli-Armstrong model [21], the artificial neural network model [22], the dislocation based model [4], the damage based model [8], etc. In the present investigation, the modified Arrhenius model was chosen to establish the stress (*σ*) and strain (*ε*) relation under various strain rates ($\dot{\varepsilon }$) and temperatures (*T*) because of the advantage in convenience and accuracy. The modified Arrhenius model was expressed as follows [23,24]:

(4) $$\dot{\varepsilon }\cdot \exp\left(\frac{Q}{RT}\right)=A{\left(\sinh\left(\alpha \sigma \right)\right)}^{n}$$

Here, *R* was the gas constant (8.314 J/(mol∙K)). *Q* was the thermal activation energy (J/mol). *n* was the stress index. *A* and *α* were material constants. In the modified Arrhenius model, *Q, A, n,* and *α* could all be expressed as a polynomial function of strain (*ε*). In the present investigation, sixth-order polynomial function was used:

(5) $$\Theta =c_{0}+c_{1}\varepsilon +c_{2}\varepsilon^{2}+c_{3}\varepsilon^{3}+c_{4}\varepsilon^{4}+c_{5}\varepsilon^{5}+c_{6}\varepsilon^{6}$$

Here, *Θ* denoted model parameters (*Q, A, n, α*); *c*_0_~*c*_5_ denoted sixth-order polynomial function coefficients. The key step for model construction was to obtain the model parameters (*Q, A, n,* and *α*) under different strains. Under a specific strain, the following calculation procedure was employed: (a) *n* was obtained by the slope of lnσ versus $\ln\dot{\varepsilon }$ curve. (b) *α* was calculated by the division of the slope of σ versus $\ln\dot{\varepsilon }$ curve and *n*. (c) *Q* was calculated by the slope of $\frac{1}{T}$ versus ln(sinh(ασ)) curve. (d) lnA was calculated by the intercept of ln(sinh(ασ)) versus $\ln\;\dot{\varepsilon }$ + *Q*/*RT* curve. The calculation was performed on *Matlab* software (R2016a). The calculation results of the coefficients in Equation (5) are shown in Table 2.

**Figure 8:**
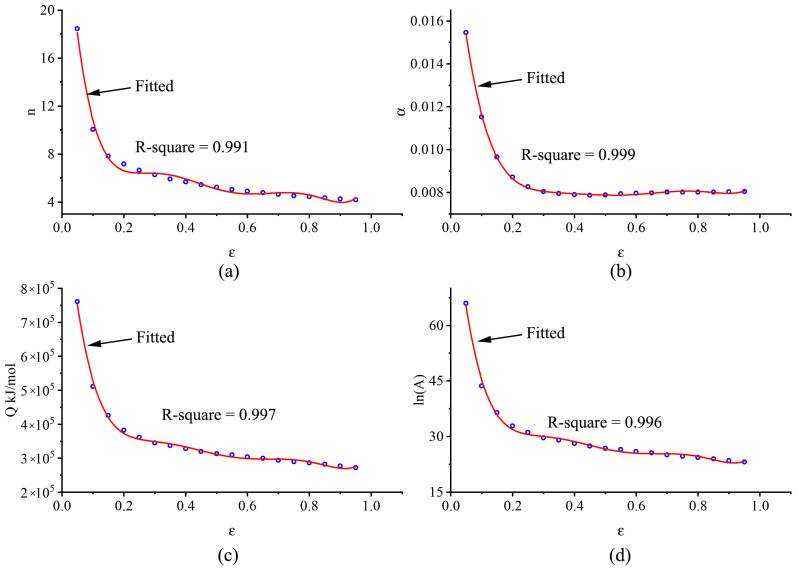
The polynomial fitting process of model parameters. (**a**) *n*, (**b**) *β*, (**c**) *Q*, (**d**) ln*A*.

The variations of model parameters with strain are shown in Figure 8. Basically, the variations of the model parameters followed a similar trend with increasing strain. When the strain was smaller than ~0.2, the stress index (*n*) decreased rapidly from 18.5 to 6.6 as the strain increased, which indicated that dislocation cross-slips and dynamic recovery were the main deformation mechanisms [25]. Meanwhile, the thermal activation energy decreased from 760.6 kJ/mol to 375.9 kJ/mol because the atom motion barrier was lowered by dynamic recovery. When the thermal activation energy dropped below the activation energy required for dynamic recrystallization, dynamic recrystallization occurred. The stress index varied between 6.6 and 4.3, indicating that recrystallization was dominant in material softening [26,27]. The thermal activation energy gradually dropped from 375.9 kJ/mol to 270.8 kJ/mol, because the thermal activation energy barrier was reduced due to the combining effect of dynamic recrystallization and dynamic recovery.

**Figure 9:**
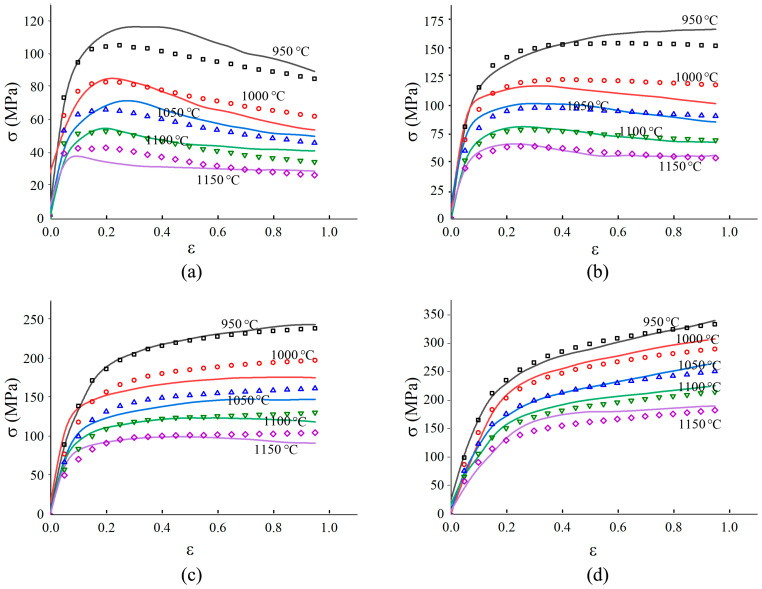
Comparisons of the calculated (dots) and experimental (lines) flow stress at the strain rates of (**a**) 0.01 s^−1^, (**b**) 0. 1 s^−1^, (**c**) 1 s^−1^, (**d**) 10 s^−1^.

A comparison was made between the experimental stresses and the calculated stresses, as shown in Figure 9. The confidence level evaluating the accuracy of the model, *R*, was expressed as:

(6) $$R=\sqrt{1-\frac{\sum_{i=1}^{n}{\left({\hat{\sigma }}_{i}-\sigma_{i}\right)}^{2}}{\sum_{i=1}^{n}{\left(\sigma_{i}-\bar{\sigma }\right)}^{2}}}$$

**Figure 10:**
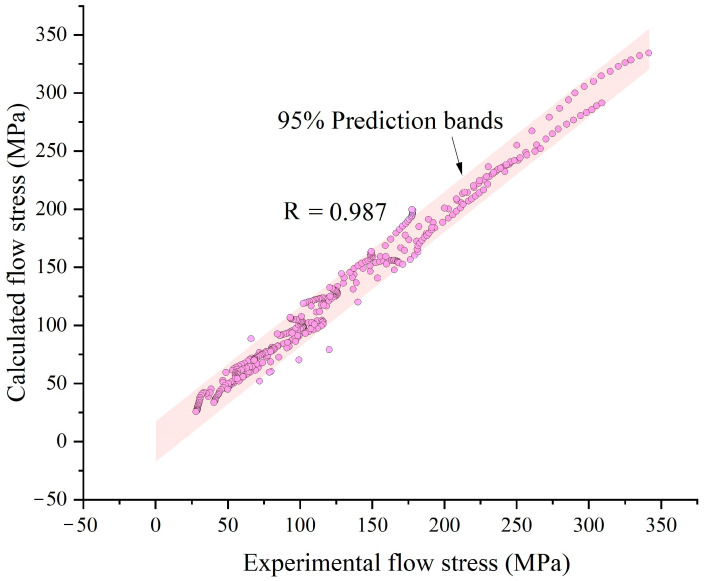
Scatter plots of the calculated flow stress versus experimental flow stress.

**Figure 11:**
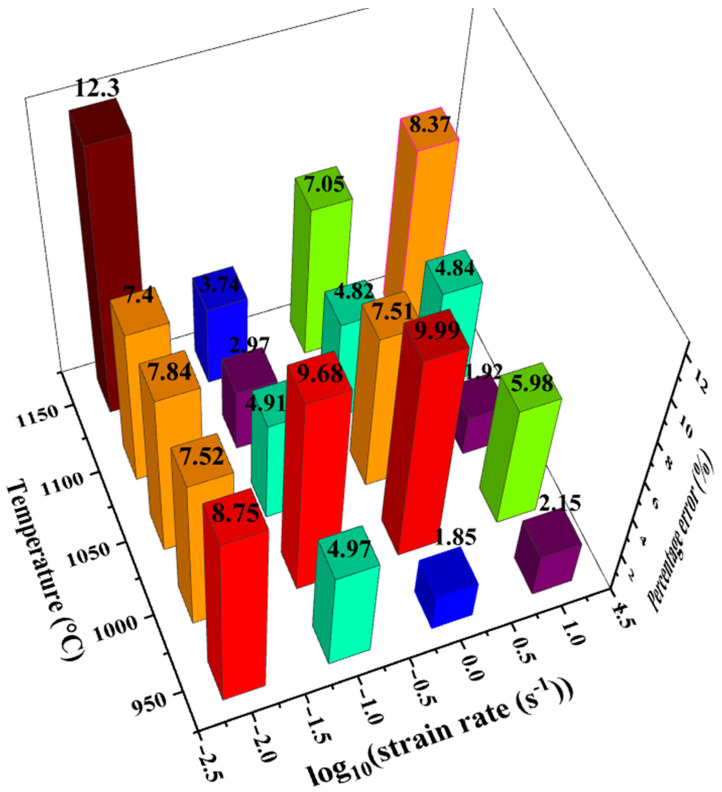
Percentage error of predicted flow stress under various tensile conditions.

Here, n was the sample number, ${\hat{\sigma }}_{i}$ the calculated flow stress of the *i*th sample, $\bar{\sigma }$ the average flow stress, and $\sigma_{i}$ the experimental flow stress of the *i*th sample. The value of *R* was calculated to be 0.987. It could also be seen that the flow stress model was able to describe both the single peaked curve shape and the exponential hardened curve shape. Figure 10 was drawn by dots whose *x* ordinates and *y* ordinates were the experimental and calculated flow stresses, respectively. Figure 11 showed the mean percentage error of model prediction under each experiment condition. The maximum percentage error (12.3%) occurred at 1150 °C and 0.01s^−1^, and the minimum error (1.85%) occurred at 950 °C and 1 s^−1^. The average error under all test conditions was 6.23% (6.81 MPa). The error was induced by polynomial fitting and experimental error. Increasing the order of polynomial fitting on the one hand improve the accuracy of the model, on the other hand, increased the number of model parameters and deter the convenience of usage of the model. Experimental error could also be decreased by increasing the number of experimental repetitions. In general, the error was acceptable, and the model was overall accurate in describing the constitutive relationship of 300M steel in isothermal tension.

## 4. Conclusions

The following conclusions could be drawn from this investigation:

(1) Under a higher temperature and a lower strain rate, the tensile force was lower and the ultimate elongation was shorter. The flow stress curve shape gradually transited from single peaked to exponential hardened when the strain rate increased.
(2) The tensile flow stresses were 29.1% (27.8 MPa) greater than the compressive flow stresses. The difference of the flow stress was caused by the difference of the stress state and the microstructure changes in dynamic recrystallization.
(3) The Arrhenius based flow stress model was able to accurately describe both the single peaked curve shape and the exponential hardened curve shape. The average deviation of the model calculation was 6.81 MPa (6.23%), and the value of *R* was 0.987.

## Figures and Tables

**Table 1:** Experimental parameters.

Test No.	Temperature(°C)	Strain Rate (s^−1^)
1	950	0.01
2	950	0.1
3	950	1
4	950	10
5	1000	0.01
6	1000	0.1
7	1000	1
8	1000	10
9	1050	0.01
10	1050	0.1
11	1050	1
12	1050	10
13	1100	0.01
14	1100	0.1
15	1100	1
16	1100	10
17	1150	0.01
18	1150	0.1
19	1150	1
20	1150	10

**Table 2:** Coefficients of the polynomials.

Coefficients	*n*	*α*	*Q*	ln*A*
***c*** **_0_**	34.414	0.022	1.167 × 10^6^	2.700
***c*** **_1_**	−380.819	−0.165	−1.081 × 10^7^	64.484
***c*** **_2_**	2198.841	0.809	5.820 × 10^7^	509.409
***c*** **_3_**	−6344.018	−2.072	−1.604 × 10^8^	1816.843
***c*** **_4_**	9592.681	2.894	2.353 × 10^8^	3222.050
***c*** **_5_**	−7269.985	−2.080	−1.746 × 10^8^	2771.555
***c*** **_6_**	2177.324	0.601	5.150 × 10^7^	921.238

## Data Availability

Data available in a publicly accessible repository.
